# Intestinal Obstruction Due to Giant Bilateral Krukenberg’s Tumors Synchronous of Colorectal Origin

**DOI:** 10.7759/cureus.14176

**Published:** 2021-03-29

**Authors:** Naveen Kumar Gaur, Oseen Shaikh, Chellappa Vijayakumar, Uday Kumbhar, Rajesh Nachiappa Ganesh

**Affiliations:** 1 Surgery, Jawaharlal Institute of Postgraduate Medical Education and Research, Puducherry, IND; 2 Pathology, Jawaharlal Institute of Postgraduate Medical Education and Research, Puducherry, IND

**Keywords:** ovary, carcinoma rectum, intestinal obstruction, krukenberg tumor

## Abstract

Giant bilateral Krukenberg tumors are rarely seen, especially causing complications due to their size. We present a 35-year-old female, diagnosed with carcinoma rectum one year back, now presented to us with intestinal obstruction features. Imaging was suggestive of features of acute intestinal obstruction. Intraoperatively, we found that the patient had bilateral giant ovarian cysts, which compressed the proximal part of the descending colon, causing the obstruction. The patient underwent bilateral excision of the ovarian cyst with diversion sigmoid colostomy. Postoperatively the patient was started on palliative chemotherapy.

## Introduction

Krukenberg tumor, the condition named after the German physician Friedrich Ernst Krukenberg who first described five cases, is a rare metastatic ovarian carcinoma histologically characterized by signet-ring adenocarcinoma with a primarily gastrointestinal source, which can occur in any age group; however, 35-40 years is the most common [1]. Most patients present with bilateral ovarian involvement, and the prognosis is abysmal, with a median survival of 14 months [2]. The stomach is the most common primary site, followed by the colon [3]. These tumors spread by the lymphatic route and are diagnosed histologically. The pathology accounts for nearly two percent of all ovarian tumors. However, in countries with a high incidence of gastric cancer, like Japan, Korea, and China, the prevalence is much higher [2]. Treatment and prognostic factors are not well established, but the prognosis is poor. This case report presents an unusual case of a Krukenberg tumor in a young female and highlights the diagnostic workup and treatment.

## Case presentation

A 35-year-old female presented to the emergency surgery unit for diffuse abdominal pain and distension for two months and increased intensity for five days. She also complained of bleeding per rectum for two months and suprapubic discomfort while urinating for two weeks.

She was diagnosed with carcinoma rectum and bilateral adnexal mass one year back. However, she did not follow up for further planning and managing the disease due to her financial conditions and non-severity symptoms. One year later, she presented to the emergency surgery department with features suggestive of intestinal obstruction.

Physical examination revealed a large, firm, tender mass occupying the entire abdomen. Abdomen radiography revealed multiple air-fluid levels. Computed tomography (CT) abdomen and pelvis showed Irregular circumferential wall thickening involving the mid and lower rectum for a length of 8 cm with a maximum wall thickness of 22 mm. The distal margin of the lesion is 3 cm from the anal verge. Anteriorly lesion showed loss of fat planes with the cervix. Proximal to the lesion, large bowel loops appeared dilated with air with a maximum caliber of 6.7 cm. A large abdominopelvic multiloculated cystic lesion with multiple thin septations and few solid enhancing areas occupying the abdominopelvic region was noted, measuring 19x16x24 cm in its axial and longitudinal planes. Bilateral ovaries were not separately visualized; hence lesion origin was possibly from the ovaries. The lesion had indistinct fat planes with the anterior abdominal wall and was displacing the small bowel loops postero-superiorly and abutting them.

**Figure 1:**
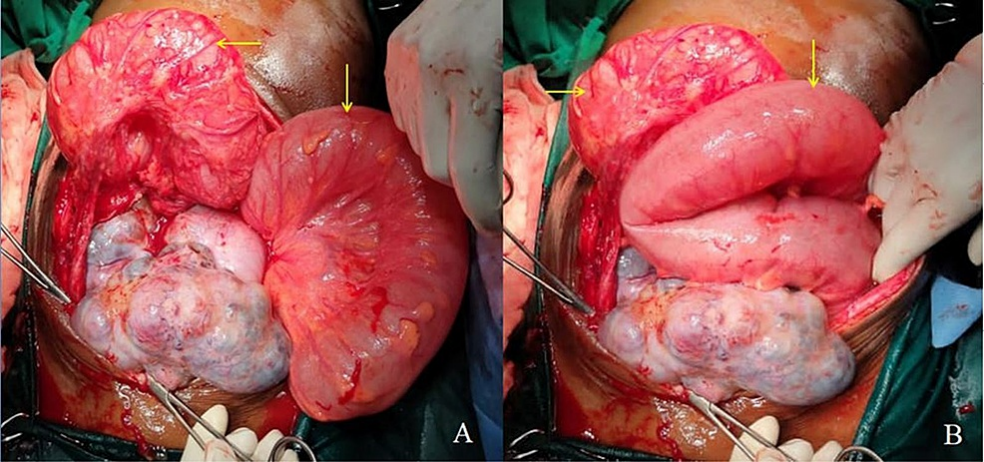
Intraoperative picture showing grossly dilated large bowel loops (A and B).

Laboratory results revealed elevated serum carbohydrate antigen 125 (CA125) levels (276 U/mL). Based on clinical, laboratory, and image analysis, she was diagnosed with an acute intestinal obstruction and was taken for emergency laparotomy. Intraoperatively we found a gross dilatation of the large bowel up to the proximal part of the descending colon and collapsed distal part of the descending colon (Figure 1).

**Figure 2:**
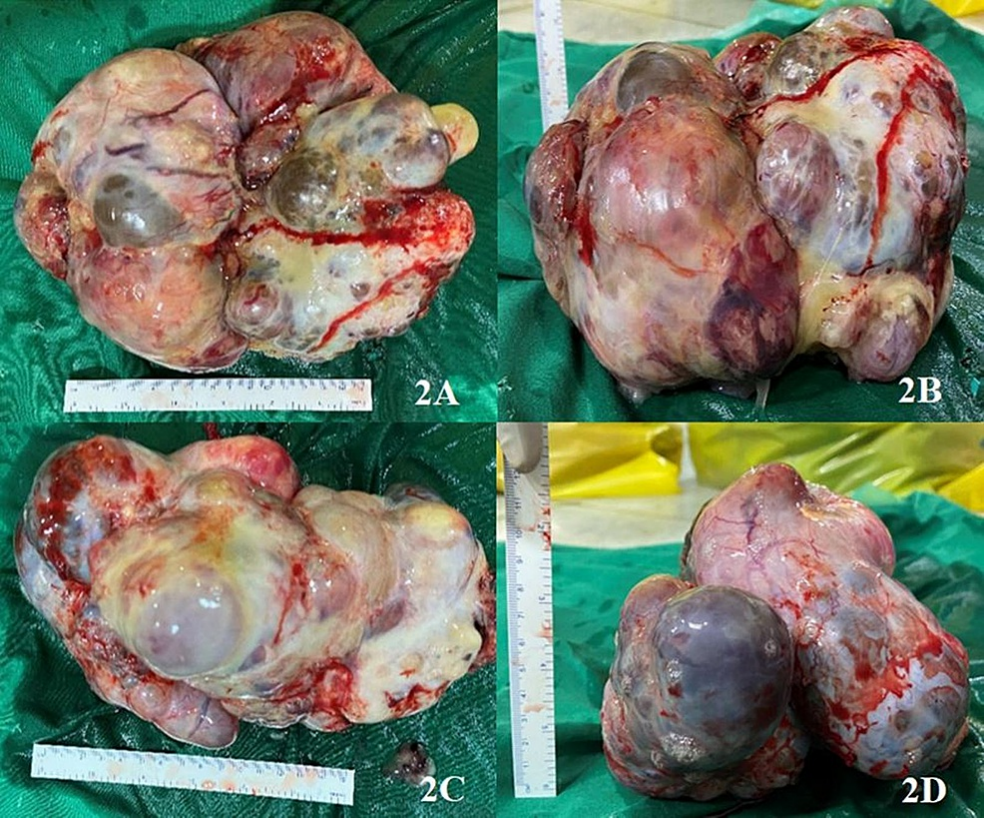
Image showing excised ovarian cysts; 2A and 2B: Right-sided ovarian cyst measuring 20x15cm, and 2C and 2D: Left ovarian cyst measuring 18x15cm.

There were two large ovarian cysts of size nearly 20x15 cm, each involving fallopian tubes compressing the descending colon's proximal part, with multiple serosal, peritoneal, omental, liver deposits. Hence palliative bilateral salpingo-oophorectomy was done with diversion loop sigmoid colostomy (Figure 2).

**Figure 3:**
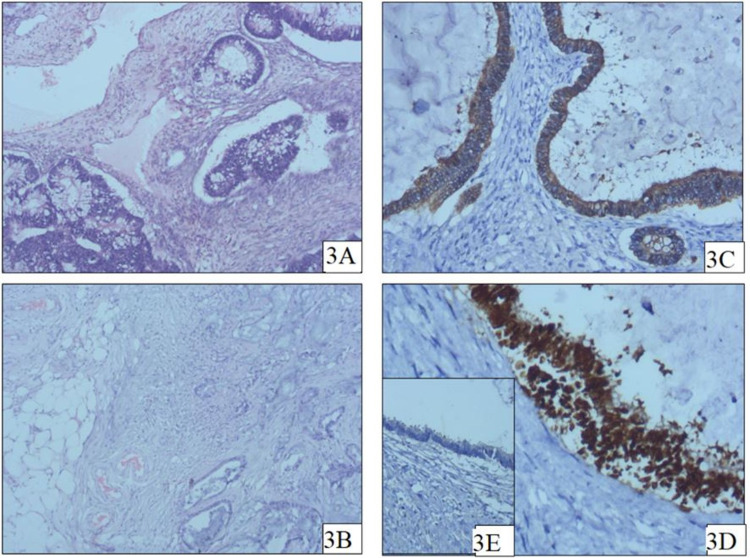
Histopathological images; 3A: Section from ovary shows infiltration by adenocarcinoma with goblet cell differentiation, 3B: Section shows omentum infiltrated by adenocarcinoma cells floating in pools of extracellular mucin, 3C: Section shows tumor cells showing prominent intracytoplasmic expression for Cytokeratin 20, a polyclonal antibody for Cytokeratin 20, 3D: Section shows tumor cells showing prominent nuclear expression for Caudal-related homeobox 2 (CDX2), 3E: Section shows tumor cells showing negative expression for Carbohydrate Antigen 125 (CA-125).

Pathological gross examination showed bilateral enlarged ovaries with a smooth external surface. On microscopy, both ovaries showed multiple cystic spaces lined by columnar to cuboidal tumor cells along with signet ring cells in mucin-filled pools. Immunostains of cytokeratin 7 (CK 7) and cytokeratin 20 (CK 20) were performed, and tumor cells were positive for CK 20 and negative for CK 7. The histopathologic findings were of the Krukenberg tumor of the ovary, and the immune profile favored a metastatic rectal carcinoma (Figure 3).

She was started on orals after three days following surgery which she tolerated well, and the stoma started moving. She was discussed in the tumor clinic board for palliative chemotherapy and was planned to start the same.

## Discussion

Krukenberg tumor constitutes nearly two percent of all ovarian tumors and contains mucin-filled signet ring cells. Almost all Krukenberg tumors are always metastatic, with few rare cases of the primary occult tumor. The stomach is the most common primary site for the Krukenberg tumor, followed by colon, appendix, and breast cancer [4,5]. The average age of presentation is usually less than 40 years [5]. According to World Health Organization, diagnostic criteria for Krukenberg tumor is based on the pathological description by Serov and Scully, which include the following features: a) Presence of stromal involvement; b) Ovarian stromal sarcomatoid proliferation; c) Presence of mucin-producing signet-ring cells for making the diagnosis of Krukenberg tumor [2].

The clinical features are abdominal pain and distension, palpable mass, loss of appetite, weight loss, menstrual cycle changes, and dyspareunia. Nearly half of the cases present with ascites which develop as a late feature. Radiologically, Krukenberg tumors typically appear as a bilateral, irregular, hyperechoic solid pattern with well-defined cystic areas producing a prominent vascular signal along the cystic wall. CT usually shows solid ovarian masses with contrast-enhancing walls, unlike in primary ovarian cancer [6]. Comparison of the CA 125 levels can be used as a follow-up marker in evaluating patients with complete tumor resection [7].

The classical gross pathological features of the Krukenberg tumor are bilateral, asymmetrically enlarged ovaries with a bosselated surface. The capsular surface is smooth without adhesions or implants. The characteristic microscopic features reveal stromal and epithelial components. The mucin-laden signet ring cells with eccentric hyperchromatic nuclei occurring in nests, cords, tubules, or acini diffusely infiltrate the mesenchymal stromal component [5,8]. Immunohistochemical testing is commonly employed to guide the physician in determining the source of ovarian carcinomas. Primary ovarian malignancy tends to be highly immunoreactive for the CK7 immunophenotype and negative for CK20.

On the other hand, metastatic gastric carcinoma is generally less positive for CK7 but shows positivity for CK20. Tumour metastasis from a colorectal source is generally negative for CK7 but positive for CK20 [4]. The metastasis route to the ovaries is mainly due to retrograde lymphatic spread as the gastrointestinal tract mucosa and submucosa have a rich lymphatic plexus and are nearby retroperitoneal lymph nodes [9]. The treatment strategy is controversial in the Krukenberg tumor. There are a few different treatment options available such as cytoreductive surgery (CRS) or debulking surgery, adjuvant chemotherapy (CT), and hyperthermic intraperitoneal chemotherapy (HIPEC). HIPEC employs thermal injury to induce heat-shock protein release from tumor cells and facilitates the cytotoxicity of chemotherapeutic medication [10]. Prognosis of the disease is abysmal and depends on the source of the metastasis with a median survival of 11 months, 21.5 months, 31 months, and 19.5 months for gastric, colorectal, breast, and other origins, respectively [11]. The prognostic factors have not been well established; however, when the primary tumor is identified after the metastasis to the ovary is discovered, the prognosis is abysmal [12]. CA 125 levels are also considered as prognostic; hence, early detection of ovarian metastasis and monitoring serum CA 125 level is helpful for patient's good outcome. Carcinoembryonic antigen (CEA) levels are not of prognostic value in patients with a colorectal primary, but may serve as an adjunct to imaging for reflecting disease response to chemotherapy.

The median overall survival from Krukenberg tumor surgery is 17 months. The median survival for patients with a colorectal primary compared to all other origins was 15 months and 38 months, respectively. The median OS was also shorter for patients younger than 50 years old [11].

## Conclusions

Krukenberg tumors are rare metastatic bilateral ovarian tumors presenting with vague abdominal symptoms. Image analysis, serum CA 125 monitoring, and histologic features aid in diagnosing. Limited treatment options and poor prognosis make Krukenberg tumor a crooked and fatal tumor. Palliative surgery for patients with Krukenberg tumors can be performed safely with acceptable complication rates. Metastasectomy is a multidisciplinary team consensus influenced by several factors, including symptoms, synchronous disease, and tumor response to chemotherapy. Palliative bilateral oophorectomy should be performed to prevent the significant risk of symptomatic contralateral ovarian involvement. In case bilateral Krukenberg tumors are already present, then palliative resection of the same prolongs the survival of the patients to some extent.
